# Draft Genome Sequence of *Ralstonia* sp. MD27, a Poly(3-Hydroxybutyrate)-Degrading Bacterium, Isolated from Compost

**DOI:** 10.1128/genomeA.01170-15

**Published:** 2015-10-08

**Authors:** Morgan Zhu, Lucy M. McCully, Mark W. Silby, Tamunonengiyeofori I. Charles-Ogan, Jean Huang, Christopher J. Brigham

**Affiliations:** a Franklin W. Olin College of Engineering, Needham, Massachusetts, USA; b Department of Biology, University of Massachusetts Dartmouth, North Dartmouth, Massachusetts, USA; c Department of Bioengineering, University of Massachusetts Dartmouth, North Dartmouth, Massachusetts, USA

## Abstract

*Ralstonia* sp. strain MD27, a novel biopolymer-degrading betaproteobacterium, was isolated from compost samples. This organism has been shown to utilize the biopolymer poly(3-hydroxybutyrate) [P(3HB)] as a carbon source for growth. We report the draft genome sequence of MD27 with an estimated total sequence length of 5.9 Mb.

## GENOME ANNOUNCEMENT

*Ralstonia* species are a diverse group that includes plant and human pathogens and other soil-dwelling organisms, such as *Ralstonia eutropha* (now known as *Cupriavidus necator*) (1), *Ralstonia solanacearum* (2), and *Ralstonia pickettii* (3, 4). *Ralstonia* and related species like *Cupriavidus necator* synthesize and mobilize poly(3-hydroxybutyrate) [P(3HB)] and other polyhydroxyalkanoate (PHA) biopolymers as part of their natural metabolism (5, 6). PHA has attracted much recent interest as a potential biobased, biodegradable alternative to petroleum-based plastics (7, 8).

*Ralstonia* sp. strain MD27 was isolated from compost from the greenhouse at Wellesley College (Wellesley, MA, USA) using media containing P(3HB) as the sole carbon source. The bacterium grows using P(3HB) with a doubling time of 3.3 h. Genomic DNA of *Ralstonia* sp. MD27 was extracted using the Wizard Genomic DNA purification kit (Promega, Madison, WI, USA). Libraries were then generated using the Nextera XT sequencing kit (Illumina, San Diego, CA, USA) and sequenced by an Illumina MiSeq at Tufts University Genomics Core Facility. We obtained 2,298,090 2 × 250-bp reads (185-fold coverage), which were assembled into 72 contigs (from 611 to 600,706 bp) using CLC Genomics Workbench version 7.5.

The draft genome sequence of MD27 comprises 5,917,320 bp (63.6% G+C content) and was annotated using the NCBI PGAP pipeline. We predicted 5,382 protein coding sequences, 47 tRNA sequences, and three copies each of 16S, 23S, and 5S rRNA genes. The 16S rDNA sequence of MD27 was found to be most closely related to that of *Ralstonia pickettii* strain 12J (99% identity based on nucleotide BLAST).

Two PHA synthase (*phaC*) genes were predicted (contigs 4 and 6), both of which appear to code for the conserved lipase box necessary for activity (9). We also detected two putative β-ketothiolase (*phaA*) genes (contigs 6 and 12), and two putative acetoacetyl-CoA reductase (*phaB*) genes (contigs 4 and 6). Indeed, the *phaCAB* sequences in contig 6 appear to form a gene cluster similar to a PHA biosynthesis operon found in *C. necator* H16 (10, 11) and *R. pickettii* strain 12D. A putative regulatory gene (*phaR*) has been identified in contig 6. These findings suggest that MD27 can synthesize intracellular polyhydroxyalkanoates.

Four putative PHA depolymerase (*phaZ*) genes were identified in the MD27 genome: three intracellular depolymerase genes (contigs 2, 6, and 22) and an extracellular P(3HB) depolymerase gene (contig 34) that were predicted, based on a BLAST search with the Depolymerase Engineering Database (DED, http://www.ded.uni-stuttgart.de) (12), to be an extracellular short-chain-length PHA depolymerase with a type I catalytic domain, as previously described (13). Experimentally validated PHA depolymerases that also fall into this category include the PhaZ from *Ralstonia pickettii* (14). *Ralstonia* sp. MD27 expresses a P(3HB) depolymerase that is capable of breaking down highly crystalline P(3HB) and is unlike the well-studied model organism of PHA homeostasis, *C. necator* strain H16 (10, 11), implying that *Ralstonia* sp. MD27 could be utilized in composting items made of P(3HB).

### Nucleotide sequence accession numbers.

This whole-genome shotgun sequencing project has been deposited in DDBJ/EMBL/GenBank under accession number LFZM00000000. The version described in this paper is the first version, LFZM01000000.

## References

[1] Riedel SL, Lu J, Stahl U, Brigham CJ 2014 Lipid and fatty acid metabolism in *Ralstonia eutropha*: relevance for the biotechnological production of value-added products. Appl Microbiol Biotechnol 98:1469–1483. doi:10.1007/s00253-013-5430-8. 24343766

[2] Yabuuchi E, Kosako Y, Yano I, Hotta H, Nishiuchi Y 1995 Transfer of two *Burkholderia* and an *Alcaligenes* species to *Ralstonia* gen. nov: proposal of *Ralstonia pickettii* (Ralston, Palleroni and Doudoroff 1973) comb. nov., *Ralstonia solanacearum* (Smith 1896) comb. nov. and *Ralstonia eutropha* Davis 1969) comb. nov. Microbiol Immunol 39:897–904. 8657018 10.1111/j.1348-0421.1995.tb03275.x

[3] Hundt K, Wagner M, Becher D, Hammer E, Schauer F 1998 Effect of selected environmental factors on degradation and mineralization of biaryl compounds by the bacterium *Ralstonia pickettii* in soil and compost. Chemosphere 36:2321–2335. doi:10.1016/S0045-6535(97)10201-6. 9566302

[4] Centers for Disease Control and Prevention (CDC) 1998 Nosocomial *Ralstonia pickettii* colonization associated with intrinsically contaminated saline solution—Los Angeles, California, 1998. Morb Mortal Wkly Rep 47:285–286. 9572669

[5] Riedel SL, Lu J, Stahl U, Brigham CJ 2014 Lipid and fatty acid metabolism in *Ralstonia eutropha*: relevance for the biotechnological production of value-added products. Appl Microbiol Biotechnol 98:1469–1483. doi:10.1007/s00253-013-5430-8. 24343766

[6] Jendrossek D 2009 Polyhydroxyalkanoate granules are complex subcellular organelles (carbonosomes). J Bacteriol 191:3195–3202. doi:10.1128/JB.01723-08. 19270094 PMC2687172

[7] Brigham CJ, Sinskey AJ 2012 Applications of polyhydroxyalkanoates in the medical industry. Int J Biotechnol Well Ind 1:53–60. doi:10.6000/1927-3037.2012.01.01.03.

[8] Brigham CJ, Sinskey AJ 2012 Polyhydroxyalkanoate production enzymes: a survey and biological perspective. J Siberian Fed Univ. 3:220–242.

[9] Rehm BH 2003 Polyester synthases: natural catalysts for plastics. Biochem J 376:15–33. doi:10.1042/BJ20031254. 12954080 PMC1223765

[10] Peoples OP, Sinskey AJ 1989 Poly-beta-hydroxybutyrate (PHB) biosynthesis in *Alcaligenes eutrophus* H16: identification and characterization of the PHB polymerase gene (phbC). J Biol Chem 264:15298–15303. 2670936

[11] Peoples OP, Sinskey AJ 1989 Poly-beta-hydroxybutyrate biosynthesis in *Alcaligenes eutrophus* H16: characterization of the genes encoding beta-ketothiolase and acetoacetyl-CoA reductase. J Biol Chem 264:15293–15297. 2670935

[12] Knoll M, Hamm TM, Wagner F, Martinez V, Pleiss J 2009 The PHA depolymerase engineering database: a systematic analysis tool for the diverse family of polyhydroxyalkanoate (PHA) depolymerases. BMC Bioinformatics 10:89. doi:10.1186/1471-2105-10-89. 19296857 PMC2666664

[13] Behrends A, Klingbeil B, Jendrossek D 1996 Poly(3-hydroxybutyrate) depolymerases bind to their substrate by a C-terminal located substrate binding site. FEMS Microbiol Lett 143:191–194. doi:10.1111/j.1574-6968.1996.tb08479.x. 8837471

[14] Hiraishi T, Hirahara Y, Doi Y, Maeda M, Taguchi S 2006 Effects of mutations in the substrate-binding domain of poly[(R)-3-hydroxybutyrate] (PHB) depolymerase from *Ralstonia pickettii* T1 on PHB degradation. Appl Environ Microbiol 72:7331–7338. doi:10.1128/AEM.01187-06. 16963553 PMC1636158

